# Variability in the Deformability of Red Blood Cells: Application to Treating Premature Newborns with Blood Transfusion

**DOI:** 10.3390/ijms26178144

**Published:** 2025-08-22

**Authors:** Dan Arbell, Alexander Gural, Gregory Barshtein, Sinan Abu-Leil, Lisandro Luques, Benny Gazer, Saul Yedgar

**Affiliations:** 1 Pediatric Surgery, Hadassah University Hospital, Jerusalem 91120, Israel; arbell@hadassah.org.il (D.A.); luques@hadassah.org.il (L.L.); gazer@hadassah.org.il (B.G.); 2 Blood Bank, Hadassah—Hebrew University Medical Center, Jerusalem 91120, Israel; gural@hadassah.org.il; 3 Department of Biochemistry, The Faculty of Medicine, Hebrew University, Jerusalem 91120, Israel; saulye@ekmd.huji.ac.il; 4 Department of Neonatology, Hadassah Mount Scopus, Hadassah—Hebrew University Medical Center, Jerusalem 91200, Israel; abu_leil@hadassah.org.il

**Keywords:** red blood cells, premature newborns, RBC transfusion, RBC storage, blood bank, RBC deformability

## Abstract

Blood units are routinely collected from adult donors and stored as packed red blood cells (PRBC). The quality of PRBC, including their deformability, decreases during storage. Since PRBC transfusion has been reported to promote circulatory issues in premature neonates (PNs), they typically receive freshly stored units. To test the hypothesis that freshly stored PRBCs can provide red blood cells (RBCs) with appropriate deformability for PN recipients, we compared the deformability of PRBCs transfused to PNs with that of cord blood RBCs (CRBCs), which are known to have deformability equivalent to that of newborn RBCs (PN-RBC). We found that, on average, CRBC deformability was higher than that of PRBCs. However, both showed significant variability with overlapping ranges. A highly significant correlation was observed between cell deformability and the combined levels of specific membrane proteins (ezrin, stomatin, flotillins) and membrane-bound hemoglobin (Pearson coefficient > 0.70, *p* < 0.02). This study indicates that the storage duration is inadequate for selecting PRBCs for PN recipients. PRBCs with deformability similar to that of PN-RBCs could enable safer and more effective transfusions for PN patients. Measuring membrane proteins alongside membrane-bound hemoglobin can serve as a useful method for selecting appropriate PRBC units for transfusion to PNs.

## 1. Introduction

Preterm birth is a significant cause of morbidity and mortality during the neonatal period. It is associated with a relatively high incidence of specific pathological conditions among premature neonates (PNs), such as intraventricular hemorrhage (IVH), necrotizing enterocolitis (NEC), bronchopulmonary dysplasia (BPD), and retinopathy of prematurity (ROP) [1,2,3,4,5]. The development of diseases related to preterm birth is multifactorial and remains poorly understood. Still, numerous studies indicate that the onset of these conditions may be triggered by the transfusion of packed red blood cells (PRBCs) collected from adult donors [6]. Up to 90% of extremely low birth weight (ELBW) newborns, and approximately 60% of PNs born before 32 weeks of gestation, are treated with PRBC transfusion during their hospital stay [7].

Retrospective studies with a large cohort of PNs have demonstrated that PRBC transfusions are independently linked to an increased likelihood of developing ROP [8], particularly among those with low birth weight, with the risk escalating alongside the volume of transfused blood and the number of transfusions [9]. A multi-hospital retrospective study [10] showed that transfusion of PRBC to PNs was associated with both the incidence and severity of BPD.

Several studies have suggested that PRBC transfusion increases the risk of developing NEC-associated pathologies among prematurely born recipients [11,12,13]. However, more recent studies found no association between PRBC transfusion and NEC development [14,15,16]. Moreover, another study [17] suggested that PRBC transfusion can protect against NEC. Accordingly, a recent review [18] summarized the findings and concluded that further research in this area is necessary.

The primary role of red blood cells (RBCs) is to facilitate the exchange of gases throughout the body. To accomplish this, RBCs have unique flow-affecting properties, including their deformability, i.e., their ability to deform under flow-induced shear stress during flow, which is essential for blood circulation [19], especially in the microcirculation. RBCs with impaired deformability may impede tissue saturation [20,21,22], and rigid RBCs can occlude capillaries [23]. The transfusion of RBCs with reduced deformability induced liver necrosis in rats [21]. In previous clinical studies, we have demonstrated that the deformability of transfused PRBCs is a significant determinant of the transfusion outcome, as indicated by the transfusion-induced changes in recipient hemoglobin level (∆Hb) [24] and skin blood flow (∆SBP) [25].

Independently, it was found that the RBC deformability is impaired during storage, starting as early as the second week of storage [26,27], implying that PNs should be given shortly after stored PRBC units [28,29]. Following this, it was proposed that the freshest blood available should be given to the smallest and youngest babies [30]. Various medical institutions have implemented age restrictions on PRBC units for transfusion to premature infants, limiting storage to 7-10 days. Following this, a previous study found that the deformability of PRBC remains at its initial level for approximately 10 days before starting to decline [26,31]. The reduction in deformability is correlated with changes in the cell membrane protein composition [32] and the quantity of hemoglobin bound to the cell membrane [33].

Key differences between newborns and adult RBC membranes [34] include the content of membrane proteins [35] and lipids [36], as well as membrane fluidity [37], cell shape [34], and osmotic fragility [38]. Of special interest is the difference in hematological indices between PNs’ and adults’ RBCs: For PNs, the mean corpuscular volume (MCV) ranges from 119 fL to 106 ± 4 fL depending on the gestational age, the mean corpuscular hemoglobin (MCH) level ranges from 40 pg to 36 pg, and the mean corpuscular hemoglobin concentration (MCHC) of 34 ± 1 g/dL [39]. In comparison, for adults’ PRBCs, stored for seven days, MCV = 90 ± 3.7 fl, MCH = 30.8 ± 1.5 pg, and MCHC = 31 ± 1.5 g/dL [34,40]. CRBCs are thus significantly larger and contain a higher amount of hemoglobin than PRBCs.

These differences have implications for blood perfusion through the capillaries, the critical zone for RBC passage, which measures 3–6 µm in diameter, for both neonates and adults [41]. Preterm infants exhibit higher Functional Vessel Density (FVD), a greater proportion of small vessels, and a lower value of vessel surface (VS), reflecting a unique microvascular phenotype [42]. Taken together, PN-RBCs require 27% to 100% higher driving pressure than adult RBCs to deform sufficiently to traverse a 4.5 µm capillary. However, when PN-RBCs are suspended in their own (neonatal) plasma, which has lower viscosity, the required pressures become similar to those of adults [43].

The unique nature of the microcirculation system in PNs highlights the crucial role of blood rheology, and particularly RBC deformability, in ensuring proper blood flow [44,45,46].

In summary,

- Preterm neonates, particularly with very low birth weight, have relatively large RBCs, leading to increased mechanical stress during their passage through the microcirculation, particularly the capillaries.
- Effective perfusion in newborns depends mainly on the lower viscosity of neonatal plasma and an increased capillary density.
- These aspects emphasize the crucial role of RBC deformability in maintaining sufficient oxygen delivery in neonatal critical care.

Indeed, in a previous small study, we showed that, in general, the deformability of RBCs collected from PNs is higher than that of the PRBCs they received [47]. We also demonstrated that RBC deformability varies significantly between donors [40], while the processing of donated blood may enhance it by removing undeformable cells [48]. In another study, we found that the deformability of cord blood RBCs (CRBCs) is equal to that of the respective PN on the day of birth [39], before any treatment or transfusion, which can affect their RBC characteristics.

On these grounds, the present study was undertaken to test the hypothesis that the use of shortly stored PRBC units for PN transfusion can secure the provision of RBCs with deformability that is higher than, or at least equal to, that of the recipients. To this end, we compared the deformability of PRBCs transfused to PNs to that of RBCs collected from the cord blood of PN recipients.

## 2. Results

### 2.1. Deformability of Red Blood Cells from PN and PRBC Units

In a previous study [47], we demonstrated that the deformability of PN-RBCs equals that of their autologous cord blood RBCs (CRBCs). Therefore, to determine the deformability of PRBCs vs. that of RBCs from PNs, we compared the deformability of RBCs isolated from the umbilical cord blood of the PNs to that of the PRBCs transfused to them.

**Figure 1:**
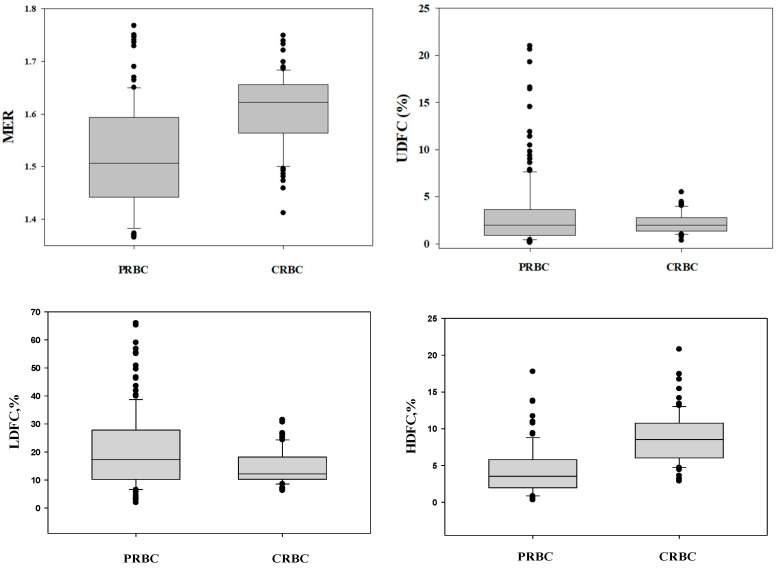
Variability in RBC deformability in packed RBCs (PRBCs, *n* = 156) and cord blood RBCs (CRBCs, *n* = 78). The detailed statistics of the data presented in Figure 1 are summarized in Table 1.

As described in the Materials and Methods Section 4.4 below, the cell flow properties image analyzer (CFA) quantifies the elongation of cells under flow-induced shear stress. It provides, for each cell, the elongation ratio ER = A/B, where A and B represent the cell’s major and minor axes, respectively. The image analysis provides the distribution of the RBC deformability in a large cell population, from which the relevant parameters are determined, as shown in Figure 1; these are derived as follows: MER = median elongation ratio; AER = average elongation ratio; %HDFC = percent of high deformable cells (ER ≥ 2.5) in the RBC population; %LDFC = percent of low deformable cells (1.1 < ER < 1.3) in RBC population; %UDFC—percent of undeformable cells (ER < 1.1) in the RBC population (for details, see the methods in Section 4.4 below).

Table 1 and Figure 1 show that, on average, the deformability of CRBCs is significantly higher than that of transfused PRBCs. However, in both CRBCs and PRBCs, the deformability varies considerably. For example, for CRBCs, the MER values ranged from 1.41 to 1.75, and the percentage of undeformable cells (UDFCs) ranged from 0.35% to 5.50%. Similarly, for PRBCs, the MER values varied between samples from 1.24 to 1.77, and the percentage of undeformable cells (%UDFC) ranged from 0.12 to 21.0.

**Figure 2:**
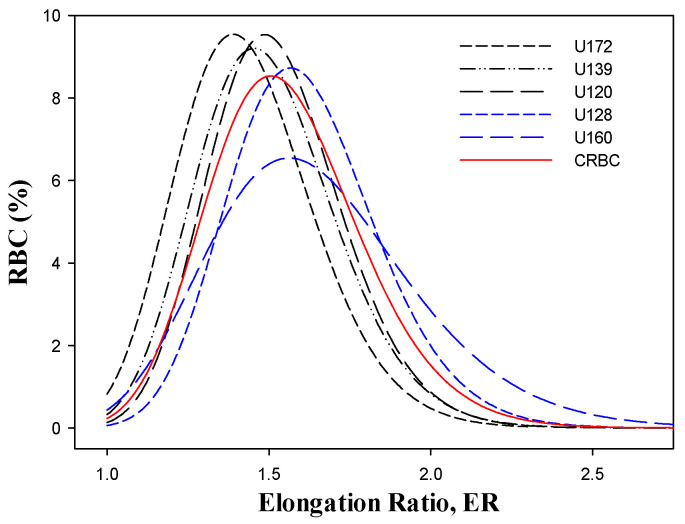
Elongation ratio (ER) distribution curves for five PRBC units transfused to one PN recipient.

The concern regarding the deformability of transfused PRBCs is further accentuated by the results presented in Figure 2 and Table 2, showing the variability in the deformability of five PRBC units transfused to one premature newborn, compared to the deformability of its own CRBCs.

It can be seen that for two CRBC units (U128 and U160), the ER distribution curves are located to the right of the CRBC curve (i.e., they have higher deformability). In comparison, those of three other PRBC units (U120, U139, and U172) are located to the left (lower deformability).

The specific data derived from the distribution curves, depicted in Table 2, show that compared to the indices of the recipient CRBC, some transfused PRBC units (U120, U139, and U172) had, as expected, lower MERs and a lower portion of highly deformable cells (%HDFC), but the opposite was observed in other PRBC units (U128 and U160).

### 2.2. Determination of RBC Membrane Proteins for Assessing Cell Deformability

Due to the variability in PRBC deformability, it seems that its determination before transfusion may be used to ensure the supply of a PRBC unit with HF that is better than that of the PN recipient. This can be performed, of course, using a rheological apparatus, such as the CFA used in the present study. An alternative approach is the biochemical approach. In previous studies, we demonstrated that RBC deformability correlates with the content of several membrane proteins [32] and the amount of hemoglobin bound to the inner side of the membrane [33]. In this study, we explored the correlation between cell deformability and specific biochemical markers, including membrane proteins and the membrane-bound hemoglobin (Hb) level, indicated by the membrane-bound β-subunit (HBB), as a potential highly reliable method for assessing deformability.

To this end, we used the following equation:

MER = a × [Protein] + b × [HBB]

where

[Protein]—the level of the protein in the RBC membrane determined by mass spectrometry (expressed by Ln(LFQ));

[HBB]—the level of the hemoglobin β-subunits bound to the RBC membrane (expressed by Ln(LFQ)).

The results, presented in Table 3, clearly show that this combination provides a highly reliable tool for the determination of RBC deformability, and can be used for personalized transfusion by selecting a PRBC unit with deformability that is higher than, or at least equal to, that of the PN recipient.

## 3. Discussion

### 3.1. Unique Characteristics of Microcirculation in Premature Neonates

The outcome of adult RBC transfusion to neonates, primarily the prematurely born, has raised increasing concerns, as both prospective studies and retrospective analysis provide evidence that it might facilitate microcirculatory pathologies (NEC in particular) in the recipients [18,49,50]. Yet, since other studies did not provide supportive data, this is not unequivocally accepted [18].

It is well-documented that the functionality of PRBCs begins to decline approximately two weeks after storage [26,27]. As in blood banking practices, storage duration is used as the sole factor that characterizes the quality of PRBCs. However, the actual functionality of individual PRBC units, specifically their capacity to produce the expected transfusion outcome, is often overlooked [51]. This applies especially to the hemodynamic functionality of PRBCs, i.e., their ability to affect recipients’ blood flow. As noted above, RBC deformability was shown to be a potent factor in PRBC transfusion efficiency as expressed by the transfusion-induced change in the recipient’s Hb (∆Hb) and skin blood flow (∆SBF) [25]: both ∆Hb and ∆SBF increased as a function of the deformability of transfused PRBCs [24,25].

Due to concerns regarding how blood banking processes and the duration of storage impact the quality of packed red blood cells (PRBCs), specific recipient groups, such as neonates or patients who require frequent transfusions (like those with hemoglobinopathies), typically receive PRBCs that have been stored for a shorter duration (less than 10 days). However, this measure alone is insufficient, as the properties of RBCs can vary significantly between different donors [52].

### 3.2. Variability in Deformability of Donors’ and Recipients’ RBCs, and Its Implications for Blood Transfusion Outcomes

Donated RBCs exhibit considerable inter-donor variability, which can affect their susceptibility to storage lesions [53,54]. For example, variations in the antioxidant activity of the donated RBCs modulate the kinetics of their storage lesion and, likely, their transfusion efficacy [54,55]. Inter-donor differences were observed in the PRBC ATP level and the post-transfusion 24 h PRBC survival in recipients’ circulation [53,56]. Similarly, it was reported [57] that at 5 days post-collection, RBC mechanical fragility exhibited up to two-fold inter-donor variability, and this was further increased during storage up to ten-fold from the initial variability. Specifically, in relation to the present study, we have previously observed considerable variability in the deformability of both freshly donated RBC and PRBC units with comparable storage durations [31].

This study validated earlier findings, revealing that the deformability of PRBCs transfused to infants, irrespective of their storage duration, varies considerably among different units (Figure 1 and Table 1). Moreover, we discovered notable variability in cell deformability for CRBCs collected from different PNs. At the same time, we found that, on average, the deformability of cord blood RBCs is superior to that of packed RBCs (Figure 1, Table 1), suggesting that transfusing newborns with PRBCs, routinely collected from adult donors, may introduce an undesirable risk to the recipients. These findings support the notion that newborns should receive CRBCs instead of adult-derived PRBCs, as suggested by several recent studies [47,58,59,60]. Yet, due to the significant variability in CBRC deformability, the use of CRBCs as is for transfusion to PNs does not ensure the supply of RBCs with hemodynamic functionality that is better than, or at least equal to, that of the PN recipient.

The results and considerations described above suggest the following:

1. The conventional supply of PRBC units according to their storage duration, even when shortly stored, is not sufficient for assuring that the hemodynamic functionality (expressed primarily by the deformability of the transfused RBCs) is better than that of the recipients’ RBCs; the RBC deformability should be specifically determined for each PRBC unit, independently of the storage duration.
2. Cord blood RBCs exhibit, on average, higher deformability than PRBCs, but with considerable variability. Therefore, their use for transfusion to premature newborns does not necessarily secure the provision of RBCs with better deformability than that of the newborn recipients.
3. Before transfusion, selection of PRBCs with deformability that is better than, or at least equal to, that of the recipients’ RBCs can be used for personalized, patient-specific blood transfusion to ensure safer and more efficient outcomes.
4. The selection of PRBCs is particularly pertinent to transfusions in neonates, as their RBC deformability is generally superior to that of PRBCs collected from adult donors. As noted above, premature neonates, especially those with very low birth weight (VLBW; <1500 g), are prone to developing pathologies (e.g., NEC, IVH, BPD, ROP), which are linked to compromised microcirculation [50,61,62], in which RBC deformability plays a major role [63,64]. High RBC deformability allows smooth passage through the capillaries and effective oxygen delivery [65]. Conversely, less deformable/rigid RBCs can exacerbate hypoxia, increase vascular resistance, and impede perfusion, potentially worsening these conditions [66,67,68,69]. Accordingly, the transfusion of RBCs with low deformability can impair the transfusion outcome, as expressed by the transfusion-induced change in Hb increment and skin blood perfusion [24,25]. Therefore, the transfusion of PRBCs with proper deformability is essential for safe and efficient RBC transfusion, thereby reducing the potential for developing circulatory pathologies.

All in all, the findings and considerations presented here imply that the functionality of PRBCs, as defined by their capacity to provide the desired transfusion outcome, only partially depends on the storage duration. At any storage duration, the PRBC functionality is the result of the initial RBC properties, which vary greatly between donors and the blood banking procedure applied to the unit, and as a result of the differential effect of storage duration. Thus, the FIFO criterion is not sufficient for assessing the potential transfusion outcome. Regardless of storage duration, the RBC flow-affecting properties should be assessed specifically for each PRBC unit and, ideally, compared to those of the recipient’s RBCs. As noted above, the current tests of donated blood are confined to immunological factors (blood typing, infectious agents). Additional testing of RBC hemodynamic quality would introduce a novel, possibly paradigm-changing practice into blood banking, offering personalized, safe, and improved transfusion therapy.

### 3.3. Future Perspectives

As stated above, we have previously shown that the loss of several proteins from the membrane and an increase in the amount of membrane-bound hemoglobin independently result in decreased RBC deformability [32,33]. As shown in Table 3, the accuracy of predicting cell deformability is considerably improved by the combined determination of the level of the membrane protein(s) and the membrane-associated hemoglobin.

RBC aging is associated with a depletion of these proteins due to the vesiculation of their membranes [70,71] and subsequent reorganization of the membrane, modifying the interaction between the lipid bilayer and the cytoskeleton [72,73]. As a result, these changes impair the cell’s capacity to deform under shear stress [32,33]. Additionally, the characteristic oxidative stress in RBCs leads to the attachment of hemoglobin molecules to the inner membrane surface. About 2–10% of intracellular hemoglobin can be bound to the membrane [74] and proportionally reduce the cell’s deformability [33].

The marked correlations between the molecular composition of the RBC membrane and the cell deformability may provide a highly reliable method for selecting a PRBC unit with deformability that is better than, or at least equal to, that of the recipient’s RBC. This can ensure safer and efficient transfusion, especially for patients with special needs, like premature neonates.

As noted above, RBC deformability can be determined by image analysis and/or the combined levels of membrane proteins and bound Hb. Both methods can provide the RBC medium elongation ratio (MER), which is a reliable predictor of the transfusion outcome [32].

The correlations between the molecular composition of the RBC membrane and the cell deformability (Table 3) may potentially be applied for safer and more efficient transfusion to PNs by selecting a PRBC unit with deformability that is better than, or at least equal to, that of the recipient.

## 4. Materials and Methods

### 4.1. Materials

Phosphate-buffered saline (PBS, pH = 7.4) and bovine serum albumin (BSA) were purchased from Biological Industries (Beit Haemek, Israel) and Sigma-Aldrich (Solon, OH, USA), respectively.

### 4.2. Preterm Newborn Population

The study population consisted of 78 preterm newborns with a birth weight of up to 1500 g, born at the Neonatology Unit of Hadassah University Hospital. The study received approval from the local Ethics Committee (approval number HMO-0336-15) and was conducted with informed consent from the mothers. Table 4 shows some characteristics of the PNs. Newborns were excluded if they were born to mothers with blood or coagulation disorders or diabetes. Additionally, newborns with congenital malformations or who developed pathological conditions were not included in the study.

### 4.3. Preparation of RBC Samples

#### 4.3.1. Packed RBC Collection

PRBC units (*n* = 156) were prepared from blood collected from healthy donors in the Hadassah Medical Center Blood Bank. PRBC samples were collected from the leftovers of packed cells in the PRBC unit bags upon completion of transfusion, following provision of informed consent following local Ethical Committee requirements (0819-20-HMO). All PRBC units used for the PNs were leukodepleted, γ-irradiated, and stored for less than 10 days in SAGM (preservation solution containing combinations of Saline, Adenine, Glucose, and Manitol) at 2–6 °C. Approximately 0.2 mL was collected from the PRBC unit bag upon completion of the transfusion.

#### 4.3.2. Cord Blood Collection

Blood samples were routinely collected into EDTA-containing tubes upon birth from the cord vein of preterm newborns in the Hadassah University Hospital Neonatology Unit, under the permit of the local Ethical Committee approval (HMO-0336-15), and with maternal consent.

#### 4.3.3. RBC Sample Preparation

To determine deformability, RBCs were isolated from the blood samples by centrifugation (500× *g* for 10 min) to remove plasma or storage solution, washed twice by centrifugation in PBS, and suspended at 1% hematocrit in PBS with 0.5% bovine serum albumin [48].

### 4.4. Determination of RBC Deformability

**Figure 3:**
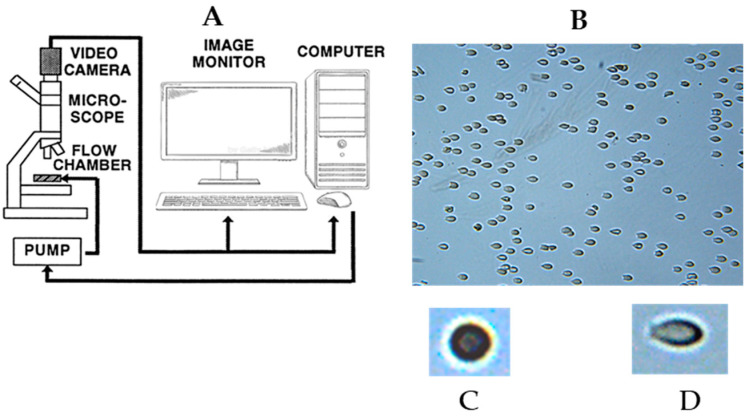
(**A**) Schematic illustration of the cell flow properties image analyzer (CFA) used for the determination of RBC deformability (see the protocol below). (**B**) Image of slide-attached RBCs under shear stress of 3.0 Pa. (**C**) Image of undeformable cell (ER = 1). (**D**) Image of highly deformable cell (ER = 2.6).

RBC deformability was determined using the computerized cell flow properties analyzer (CFA), designed and developed in S. Yedgar’s laboratory. In this image analyzer, the RBCs’ spatial and temporal shape change and dynamic organization are directly visualized in a narrow-gap flow chamber under controlled flow conditions, mimicking those in a microvessels, as previously described [25,26,32,48]. In brief, 50 μL of RBC suspension is introduced into the flow chamber (adjusted to a 200 μm gap) containing an uncoated slide. The RBCs adhering to the slide surface are subjected to a controlled flow-induced shear stress (Figure 3B) of 3.0 Pa (at 25 °C), and the change in cell shape is measured (Figure 3C,D). The CFA quantifies the cell’s elongation under flow-induced shear stress and provides, for each cell, the elongation ratio ER = A/B, where A and B represent the cell’s major and minor axes, respectively.

**Figure 4:**
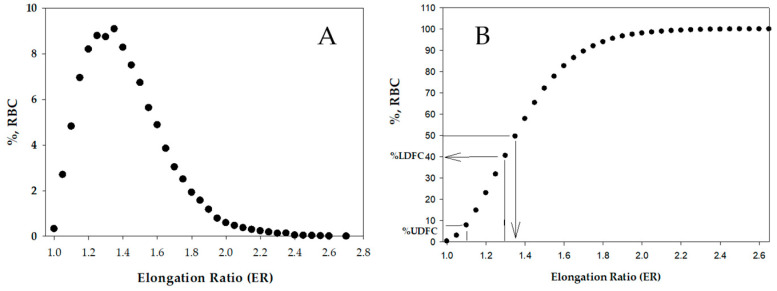
Elongation ratio (ER) distribution obtained for CRBCs. Distribution curve (**A**) and cumulative distribution (**B**). The right panel exemplifies the derivation of deformability parameters (e.g., MER and %LDFC) from the distribution curve.

An ER of 1 (Figure 3C) indicates round RBCs, namely undeformed (rigid) under the applied shear stress. Images of 25 to 35 randomly selected fields (with an area of 0.1 mm^2^) are captured and analyzed for the cell’s ER. The image analysis yields the ER distribution in 10,000 to 15,000 cells. Figure 4 presents typical ER distributions (histogram and cumulative) obtained for CRBCs. From the cumulative distribution, a series of deformability parameters are derived, including the median ER (MER) and the percentage of relatively rigid, low-deformable cells (%LDFC, ER ≤ 1.3) and undeformable cells (%UDFC, ER ≤ 1.1) [26,48]. The latter is particularly significant, as it better reflects the capacity of RBCs with reduced deformability to impair blood circulation [31].

### 4.5. Determination of RBC Membrane Protein Composition

#### 4.5.1. Preparation of RBC Membranes

One hundred microliters of washed cells (prepared in Section 4.3.3) from 15 units of PRBC was used to prepare the membranes. Cells were lysed in lysis buffer (5 mM sodium phosphate pH 8.0, 1 mM EGTA, 1 mM EDTA, and 1 mM PMSF) and placed on ice for 10 min. The hemolyzed RBC suspension was centrifuged (14,000 rpm for 10 min), and the pellet was washed three times to obtain clean RBC membranes, re-suspended in the buffer, and supplemented with a protease inhibitor cocktail to inhibit serine, cysteine, acid proteases, and aminopeptidases (P8340, Sigma, St. Louis, MO, USA). Samples were sent in dry ice for mass-spectrometry analysis.

#### 4.5.2. Determination of Membrane-Bound Hb and Membrane Protein Content

The composition of the membrane-bound Hb subunits and their levels were comprehensively determined using mass spectroscopy [75,76]. Proteins were trypsin-digested following the in-solution digestion protocol. Peptides were then purified on C18 StageTips (Fisher Scientific, Hampton, NH, USA) before their LC-MS analysis. Peptides were separated on an Easy-spray pepmap column using a water/acetonitrile gradient and the EasynLC1000 nanoHPLC (Thermo Scientific, Waltham, MA, USA). Peptides were electrosprayed into a Q-Exactive mass spectrometer (Thermo Scientific, Waltham, MA, USA) via the Easy-spray source. Peptides were analyzed using a data-dependent acquisition, with the fragmentation of the top 10 proteins from each scan. Raw MS files were analyzed by MaxQuant using the Human UniProt database (Universal Protein Resourse, Washington, DC, USA). The false discovery rate was set to 1% at the protein and peptide levels. Mass spectrometry identified and quantified 752 proteins and their levels, and 6 Hb subunits and their levels.

### 4.6. Statistical Analysis

The Shapiro–Wilk test was used to verify the normality of the continuous variables’ distribution. We found that for some deformation parameters, the distribution was not normal (see data in the Supplementary Material). However, when the sample size exceeds 30–40, the *t*-test can be applied to a non-normal distribution as well [77]. The sample size of the present study markedly exceeded this limit for both groups (156 for PRBCs and 78 for CRBCs).

The results are presented as mean ± SD and were tested for statistical significance using the Student *t*-test. Statistical differences examined with the SPSS 21 software package were considered significant at *p* < 0.05. The Pearson coefficient and *p*-value characterize the significance of linear regression between the tested parameters.

## 5. Conclusions

The data and considerations presented in this study suggest the following:

1. The conventional FIFO criterion is not sufficient for ensuring an optimal transfusion outcome, and the hemodynamic functionality of PRBCs, as expressed by the flow-affecting properties of RBCs, should be precisely determined for each PRBC unit, independently of the storage duration.
2. Cord blood RBCs exhibit, on average, better hemodynamic functionality than PRBCs, but this does not always guarantee the provision of RBCs with better deformability than that of the newborn recipients.
3. Comparison of the PRBCs’ deformability to that of the intended blood recipient’s cells can be used for personalized, patient-specific transfusion, with more efficient outcomes. This is particularly pertinent to the transfusion to neonates, as their RBC deformability is generally superior to that of adult RBCs.
4. The correlations between the molecular composition of the RBC membrane and the cell deformability (Table 3) may potentially be applied for safer and more efficient transfusion to PNs by selecting a PRBC unit with deformability that is better than, or at least equal to, that of the recipient.

Taken together, the findings and considerations of this study support the conclusion that storage duration is merely one factor, and not necessarily the primary one, determining the functionality of PRBCs, especially regarding their hemodynamic functionality. Therefore, regardless of storage duration, the hemodynamic functionality, expressed by the flow-affecting properties of RBCs, should be specifically assessed and applied to enhance the safety, effectiveness, and personalization of transfusion therapy. Additionally, employing molecular techniques such as mass spectrometry or antibody diagnostic kits to analyze RBC membrane composition could streamline the selection of the best blood unit for a premature recipient.

## Figures and Tables

**Table 1:** Variability in deformability of transfused packed RBCs (PRBCs) vs. that of cord blood RBCs (CRBCs).

Parameters	Mean ± SD		*p* Value	PRBC		CRBC	
	PRBC	CRBC		Max	Min	Max	Min
MER	1.52 ± 0.11	1.61 ± 0.07	1.34 × 10^−12^	1.77	1.24	1.75	1.41
%HDFC	4.22 ± 3.38	8.51 ± 3.64	1.60 × 10^−14^	17.77	0.30	20.8	1.90
%LDFC	20.70 ± 13.9	14.10 ± 5.52	2.8 × 10^−6^	66.00	2.0	31.5	6.3
%UDFC	3.18 ± 3.83	2.21 ± 1.06	0.019	21.00	0.12	5.50	0.35

MER = median elongation ratio; HDFC = highly deformable cells (ER ≥ 2.5); LDFC = low-deformable cells (1.1 < ER 1.3); UDFC = undeformable cells (ER < 1.1). For details, see Section 4.4 below.

**Table 2:** Variability in the deformability of five PRBC units transfused to one PN patient for 32 days.

Parameters	U120	U128	U139	U160	U172	CRBC
MER	1.52	1.60	1.49	1.63	1.43	1.55
%HDFC	4.74	7.41	4.08	13.82	2.09	6.14
% LDFC	16.40	9.73	21.33	15.25	30.53	14.48
%UDFC	1.83	1.21	2.87	2.54	3.94	1.12

**Table 3:** Correlations of the level of membrane-bound HBB subunit combined with the level of membrane proteins (determined by mass spectrometry) with the RBC deformability, expressed by the average elongation ratio (AER, determined by the CFA). The Pearson coefficient (R) and *p*-value characterize the significance of linear regression between the tested parameters.

Protein	R	*p*-Value
HBB	0.618	0.019
Ezrin	0.626	0.013
Stomatin	0.510	0.053
Band 4.1	0.552	0.035
Flotillin 2	0.571	0.032
Flotillin 1	0.632	0.015
HBB and Ezrin	0.702	0.017
HBB and Stomatin	0.706	0.016
HBB and Band 4.1	0.73	0.01
HBB and Flotillin 2	0.76	0.009
HBB and Flotillin 1	0.77	0.007

Membrane protein levels were determined by mass spectrometry (expressed by Ln(LFQ). HBB is the membrane-bound hemoglobin β-subunit; ezrin, stomatin, band 4.1, and flotillin 1 and 2 are membrane proteins.

**Table 4:** Characteristics of premature newborns.

Parameter	Value, Av ± S.D
Gestation age, weeks	25.47 ± 1.31
Birth weight, g	752 ± 157
Gender, F/M	38/40

## Data Availability

The data presented in this study are available upon request from the corresponding author.

## Supplementary Materials

The following supporting information can be downloaded at https://www.mdpi.com/article/10.3390/ijms26178144/s1.

## Abbreviations

The following abbreviations are used in this manuscript:

RBC	Red blood cell
CRBC	RBC from cord blood
PRBC	Packed red blood cell
EGTA	Ethylene glycol tetraacetic acid
EDTA	Ethylenediaminetetraacetic acid
PMSF	Phenylmethylsulphonyl fluoride
BSA	Bovine serum albumin
Hb	Hemoglobin
HBB	Hemoglobin β-subunits
FIFO	First-in first-out
SBF	Skin blood flow
PN	Premature newborn
ELBW	Extremely low birth weight
NEC	Necrotizing enterocolitis
ROP	Retinopathy of prematurity
BPD	Bronchopulmonary dysplasia
CFA	Cell flow analyzer
ER	Elongation ratio
MER	Median elongation ratio
LDFC	Low-deformable cell
UDFC	Undeformable cell
HDFC	High-deformable cell
