# Supplementary material for: Variability in the Deformability of Red Blood Cells: Application to Treating Premature Newborns with Blood Transfusion
Source: Int J Mol Sci. 2025 Aug 22;26(17):8144. doi: 10.3390/ijms26178144 (PMC12428727; doi:10.3390/ijms26178144)
Supplement: Supplementary file 1 [file ijms-26-08144-s001.zip › ijms-3773140-supplementary.pdf]

**Supplementary Materials**

Variability in the Deformability of Red Blood Cells: Application to Treating Premature Newborns with Blood Transfusion

Dan Arbell et al.

**Shapiro-Wilk test of data normality.**

**Shapiro-Wilk Test for PRBC (Number of samples is 156)**

| Parameters | MER | UDFC,% | LDFC, % | HDFC, % |
|---|---|---|---|---|
| **Mean** | 1.515 | 3.179 | 20.74 | 4.217 |
| **St.D.** | 0.112 | 3.828 | 13.898 | 3.358 |
| **Variance** | 0.013 | 14.654 | 193.163 | 11.273 |
| **Excess Kurtosis** | -0.099 | 7.908 | 1.244 | 2.164 |
| **Calculated Shapiro-Wilk statistic W:** | 0.9881 | 0.6797 | 0.9042 | 0.8663 |
| **Calculated Shapiro-Wilk p-value** | 0.1987 | 0.0000 | 0.0000 | 0.0000 |
| **Critical value of W (5% significance level):** | 0.9831 | 0.9831 | 0.9831 | 0.9831 |
| **Null Hypothesis** | **Accept** | **Reject** | **Reject** | **Reject** |

**Shapiro-Wilk Test for CRBC (Number of samples is 78)**

| Parameters | MER | UDFC,% | LDFC, % | HDFC, % |
|---|---|---|---|---|
| **Mean** | 1.606 | 2.364 | 14.53 | 8.452 |
| **St.D.** | 0.071 | 1.421 | 5.938 | 3.603 |
| **Variance** | 0.005 | 2.018 | 35.254 | 12.986 |
| **Excess Kurtosis** | -0.219 | 5.602 | 0.286 | 0.947 |
| **Calculated Shapiro-Wilk statistic W:** | 0.9738 | 0.8204 | 0.9076 | 0.9636 |
| **Calculated Shapiro-Wilk p-value** | 0.1168 | 0.0000 | 0.00004 | 0.0283 |
| **Critical value of W (5% significance level):** | 0.9677 | 0.9677 | 0.9677 | 0.9677 |
| **Null Hypothesis** | **Accept** | **Reject** | **Reject** | **Reject** |
